# Quantitative proteomics analysis of an ethanol- and a lactate-producing mutant strain of *Synechocystis* sp. PCC6803

**DOI:** 10.1186/s13068-015-0294-z

**Published:** 2015-08-05

**Authors:** Orawan Borirak, Leo J de Koning, Aniek D van der Woude, Huub C J Hoefsloot, Henk L Dekker, Winfried Roseboom, Chris G de Koster, Klaas J Hellingwerf

**Affiliations:** Molecular Microbial Physiology, Swammerdam Institute for Life Sciences, and Netherlands Institute for System Biology, University of Amsterdam, Science Park 904, 1098 XH Amsterdam, The Netherlands; Mass Spectrometry of Biomacromolecules, Swammerdam Institute for Life Sciences, University of Amsterdam, Amsterdam, The Netherlands; Photanol B.V., Science Park 408, 1098 XH Amsterdam, The Netherlands; Biosystems Data Analysis, Swammerdam Institute for Life Sciences, University of Amsterdam, Amsterdam, The Netherlands

**Keywords:** Synthetic biology, Metabolism, Pyruvate decarboxylase, Alcohol dehydrogenase, Lactate dehydrogenase, CRISPR/CAS system, Optimization of product formation

## Abstract

**Background:**

This study aimed at exploring the molecular physiological consequences of a major redirection of carbon flow in so-called cyanobacterial cell factories: quantitative whole-cell proteomics analyses were carried out on two ^14^N-labelled *Synechocystis* mutant strains, relative to their ^15^N-labelled wild-type counterpart. Each mutant strain overproduced one specific commodity product, i.e. ethanol or lactic acid, to such an extent that the majority of the incoming CO_2_ in the organism was directly converted into the product.

**Results:**

In total, 267 proteins have been identified with a significantly up- or down-regulated expression level. In the ethanol-producing mutant, which had the highest relative direct flux of carbon-to-product (>65%), significant up-regulation of several components involved in the initial stages of CO_2_ fixation for cellular metabolism was detected. Also a general decrease in abundance of the protein synthesizing machinery of the cells and a specific induction of an oxidative stress response were observed in this mutant. In the lactic acid overproducing mutant, that expresses part of the heterologous l-lactate dehydrogenase from a self-replicating plasmid, specific activation of two CRISPR associated proteins, encoded on the endogenous pSYSA plasmid, was observed. RT-qPCR was used to measure, of nine of the genes identified in the proteomics studies, also the adjustment of the corresponding mRNA level.

**Conclusion:**

The most striking adjustments detected in the proteome of the engineered cells were dependent on the specific product formed, with, e.g. more stress caused by lactic acid- than by ethanol production. Up-regulation of the total capacity for CO_2_ fixation in the ethanol-producing strain was due to hierarchical- rather than metabolic regulation. Furthermore, plasmid-based expression of heterologous gene(s) may induce genetic instability. For selected, limited, number of genes a striking correlation between the respective mRNA- and the corresponding protein expression level was observed, suggesting that for the expression of these genes regulation takes place primarily at the level of gene transcription.

**Electronic supplementary material:**

The online version of this article (doi:10.1186/s13068-015-0294-z) contains supplementary material, which is available to authorized users.

## Background

The steep increase in the use of fossil carbon during the past 200 years has generated worries about the increasing CO_2_ content of the earth’s atmosphere and its presumed consequences, like global warming and ocean acidification [1–3]. For this reason it is of utmost importance to develop new methodology and systems that will allow substitution of fossil carbon for renewable carbon, preferably driven by renewable, solar, energy so that in due time the global carbon cycle can be brought close(r) to a closure. Such systems indeed are under development and are mostly referred to as solar biofuel production systems, although much more than only liquid fuel (carriers) can be produced: for many chemical commodities and fine chemicals proof of principle for their production, based on the same approach, meanwhile has also been provided [4–7].

This development has taken place through first- to fourth-generation approaches [8–11], driven by increasing concerns about sustainability, and more and more supported by life cycle analyses [12–14]. Whereas the first generation approach would use key products from the food sector, like sucrose or starch, to produce ethanol [8, 15], in the fourth generation approach—also referred to as ‘direct conversion’—one aims at the direct conversion of CO_2_ into the desired product, without the need to form biomass (and all the minerals required to form it) as an obligatory intermediate [6, 7, 9–11]. This latter approach preferably uses cyanobacteria, to engineer them towards highly efficient ‘production systems’ for the desired product, because these organisms combine the highest efficiency in oxygenic photosynthesis [16] with straightforward application of synthetic systems biology, both in terms of molecular genetic intervention and in terms of the required computational analyses (e.g. review [17]).

Indeed, for such engineering several successful examples have meanwhile been documented, like, e.g. for ethanol, iso-butanol, and lactic acid, with product titres ranging from several tens of mM up to 0.1 M [7, 18, 19]. Engineered strains that can carry out a particular conversion efficiently, so that more than 50% of the fixed carbon is directly channelled into product, are referred to as ‘cell factories’ for that particular substrate [7]. Such engineering, however, may impose quite some stress on the producing organism, which occasionally is visible as an increased genetic instability of a production strain [7, 20–22]. This stress may originate from two different factors: first the massive re-direction of the carbon flow through the cell’s intermediary metabolism may cause metabolic adjustment that may have the characteristics of as a stress response. Second, the product titre may increase to levels that change the physico-chemical conditions of the growth medium and/or the cell, such that it causes growth inhibition.

The effect of the latter type of stress on cyanobacteria has already extensively investigated through transcriptomics and proteomics studies of the effect of the extracellular addition of an end product, e.g. ethanol and butanol, to batch cultures of particularly *Synechocystis* sp. PCC6803 (hereafter *Synechocystis*) [23–26]. Multiple stress response mechanisms were reported upon addition of either of these end products, including up-regulation of heat shock proteins, modification of the cell membrane and cell mobility, as well as induction of the oxidative stress response [23–26]. It should be noted, however, that the highest product titres so far obtained with cyanobacterial cell factories (see above) hardly cause any stress on the wild-type organisms in terms of growth retardation [27]. But one has to keep in mind that this type of end-product stress may differ between situations in which the stressor is produced intracellularly, or added in the extracellular compartment. Notably, a recent transcriptome study of prolonged ethanol production in *Synechocystis,* yielding a final level of 4.7 g/L ethanol (i.e. 2.5-fold less than the concentration of ethanol used in [23, 24] to stress the cells), showed that this product formation causes only minor changes in the level of gene expression [28].

Significantly fewer studies have been published on the physiological consequences, i.e. stress, of major rechanneling of intermediary metabolism in the ‘cell factories’. One consequence of the engineering of a high-capacity carbon sink in cyanobacteria, however, has already been noted, i.e. the increased rate of cellular CO_2_ fixation [6, 29, 30]. Here we explore the consequences of this approach (i.e. engineering of a high-capacity product-forming pathway into cyanobacteria) with a detailed proteomics analysis for cell factories for ethanol (with the two necessary heterologous genes integrated in the hosts’ chromosome) and lactic acid (with partial expression of the *ldh* gene from an exogenous plasmid). These two mutants were selected because they represent the cell factories for which we have achieved the highest carbon partitioning coefficient (between new cells and product). The results obtained show that for the ethanol-producing mutant, diverting up to 60–70% of the fixed carbon into product [31] causes little notable stress response, but rather a physiological accommodation in the form of an induction of the carbon concentrating mechanism, the CO_2_-fixing enzyme RuBisCO, and additional enzymes involved in the Calvin cycle. The highest levels achievable of carbon partitioning into lactic acid [30] did not lead to a similar increase in abundance of Calvin cycle enzymes. This high lactate productivity required introduction of a plasmid encoded lactate dehydrogenase. In this strain this elicits next to some physiological adaptation, a significantly increased expression of CRISPR associated proteins.

## Results and discussion

### Physiological analysis of product formation, i.e. ethanol and lactic acid, on *Synechocystis* sp. PCC6803

In order to determine the consequences of high rates of (intracellular) product formation (i.e. of ethanol and of lactic acid) in *Synechocystis*, the recombinant *Synechocystis* strains SAA012 [31] and SAW041 [30] were selected. The ethanol-producing strain carries pyruvate decarboxylase, *pdc,* and alcohol dehydrogenase, *adhII,* from *Zymomonas mobilis* under control of the endogenous promoter, *psbA2* [5]. The lactic acid-producing strain (i.e. SAW041) harbors a lactate dehydrogenase, *ldh,* from *Lactococcus lactis,* and a pyruvate kinase from *Enterococcus faecalis,* each under control of the strong constitutive promoter, *trc2,* with additional expression of the lactate dehydrogenase from an exogenous plasmid [32] (see “Methods”; Additional file 1: Table S1).

**Fig. 1:**
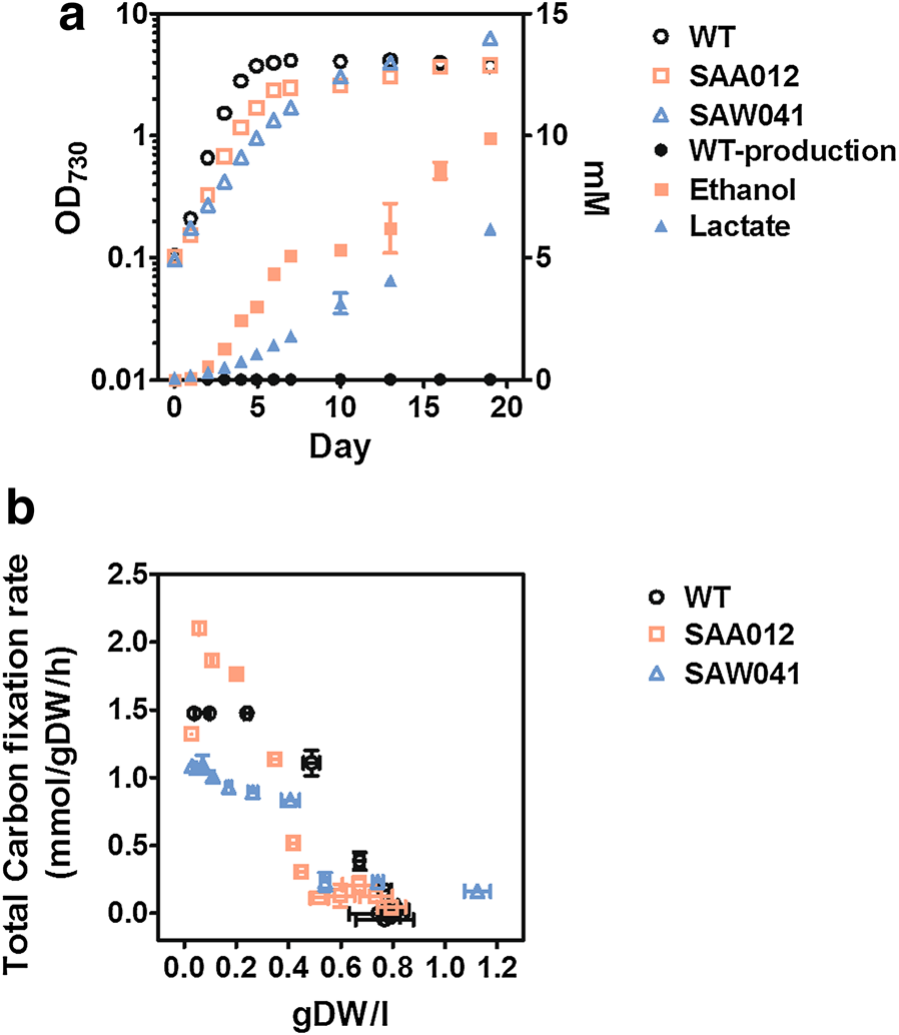
Growth, product formation, and calculated total rates of carbon fixation of the two product-forming mutants, compared to the wild-type strain *Synechocystis* sp. PCC6803. **a** Growth and product formation of the wild-type (WT) and the two product-forming strains (SAA012 and SAW041). **b** Total carbon fixation rate of SAA012, SAW041, and the corresponding WT *Synechocystis* strain plotted against the cell density of the culture. Data are mean ± SD of three biological replicates.

The two product-forming strains both grew considerably slower than the wild-type (WT) strain, which was to be expected in view of the large amount of carbon directly channeled into product. Maximum specific growth rates (*µ*) of SAA012 and SAW041 were reduced to 71 and 58% of the value of WT, respectively. The ethanol production observed in SAA012 was 9.88 ± 0.16 mM over 19 days, representing a maximum production rate of 1.33 ± 0.12 mmol l^−1^ day^−1^, while in the same period the lactic acid accumulated up to 6.19 ± 0.12 mM, with a rate of 0.36 ± 0.01 mmol l^−1^ day^−1^ (see Fig. 1a). From these physiological data the total carbon fixation rate (*q*_CO2_) was calculated (i.e. for conversion into biomass and into product). Due to the differences in growth rate between the strains (and the consequences this has on the degree of light saturation in the cultures), the *q*_CO2_ is here plotted as a function of biomass density in gDW l^−1^ (Fig. 1b). During exponential growth of the cells, when OD_730_ < 1 (i.e. up to approximately 0.2 gDW l^−1^), SAA012 exhibits a higher *q*_CO2_ than both WT and SAW041. The average increase in the *q*_CO2_ calculated was ~1.5-fold higher than the rate of CO_2_ fixation in the WT. In contrast, the lactic acid-forming strain SAW041 showed a small decrease in overall *q*_CO2_ at this cell density.

### Quantitative proteomic analysis in *Synechocystis*

To investigate the physiological consequences of intracellular product formation in the form of ethanol and lactic acid on the cellular protein composition, cells were harvested at the mid-logarithmic growth phase to minimize interference by changes in the cellular protein composition induced by the (poorly defined) factors that limit exponential growth of these cells in the transition to stationary phase. Accordingly, cells of the WT, SAA012 and SAW041 were harvested at OD_730_ = 0.7, 0.7, and 0.4, respectively (see Fig. 1a). Reference cells were harvested at OD_730_ = 0.7 from a wild-type culture for which Na^15^NO_3_ was used as the nitrogen source.

Cells from three independent cultures of the WT and of each of the two product-forming strains were harvested and mixed with the reference cells based on their OD_730_. Normalization of the ^14^N/^15^N ratio, to allow correction for possible errors in mixing the ^14^N and ^15^N samples, was performed using the median value, as described previously [33] (see also “Methods”). Upon subsequent analysis of these samples with LC-FT-MS/MS, 761, 826, and 881 proteins were quantified in WT, SAA012, and SAW041, respectively. This has resulted in a total of 1,039 unique protein ratios (and thus 716 and 633 proteins that were identified in both product-forming strains and among the three strains, respectively). The list of proteins and their respective ratio(s) quantified in this study are provided in Additional file 1: Table S2, together with their mascot scores.

**Fig. 2:**
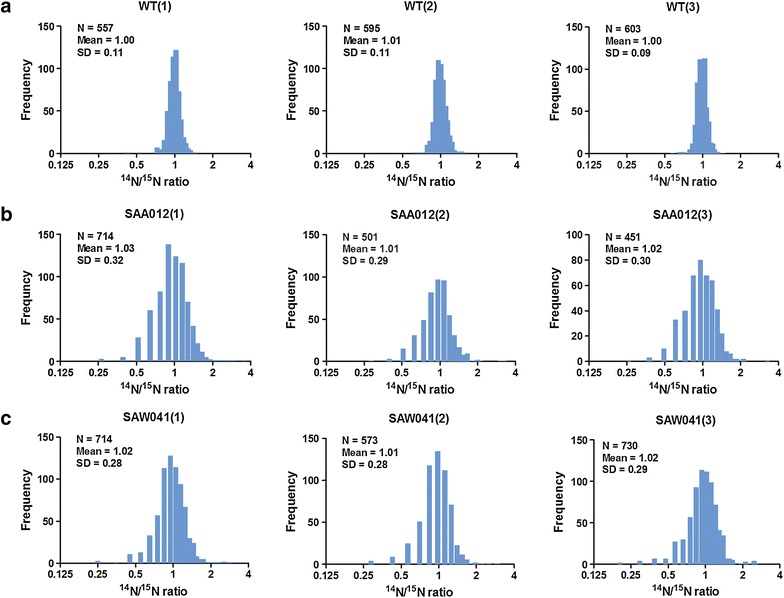
Normal distribution of normalized ^14^N/^15^N isotope ratios of quantified proteins in three biological replicates of the wild-type (WT) strain (**a**), the ethanol-producing mutant, SAA012 (**b**), and the lactate-producing mutant, SAW041* (**c**). The *abscissa* represents the number of quantified proteins (expressed as frequency), while the ordinate indicates the protein ^14^N/^15^N isotopic ratio (l/h). The number of quantified proteins, the mean value of the protein ratios, and the standard deviation of the protein ratios are indicated in the *insets*. (*Three proteins with a ratio >8-fold change were excluded).

**Fig. 3:**
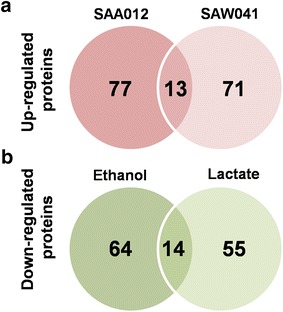
Number of differentially expressed proteins in the two mutants compared to the wild-type strain. **a** Number of significantly up-regulated proteins (*α* < 0.01). **b** Number of significantly down-regulated proteins (*α* < 0.01).

**Fig. 4:**
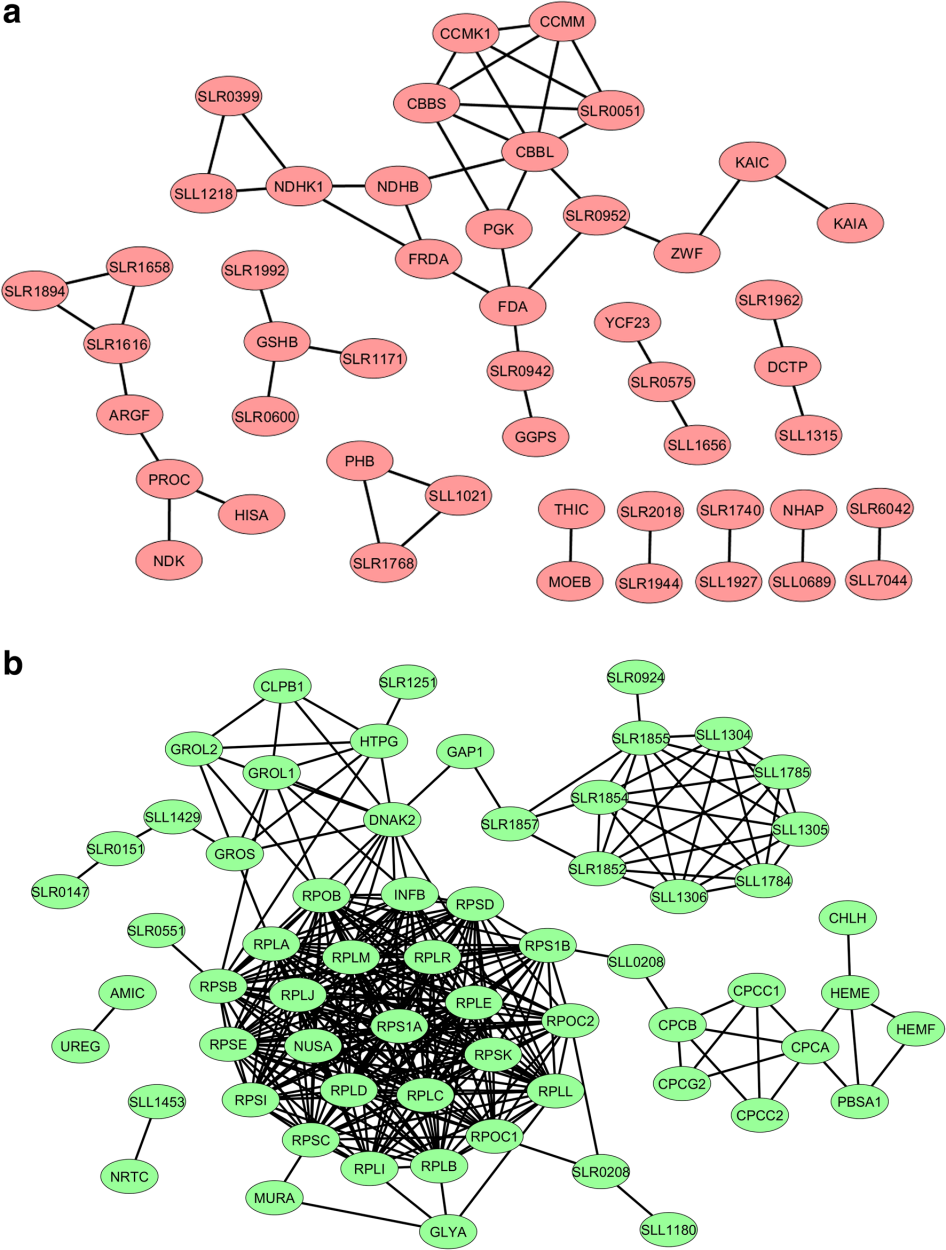
Predicted interaction of differentially expressed proteins identified in the ethanol-producing mutant, SAA012. Protein interaction was analyzed and reconstructed using STRING and Cytoscape, respectively. **a** Up-regulated proteins (*α* < 0.01). **b** Down-regulated proteins (*α* < 0.01).

**Fig. 5:**
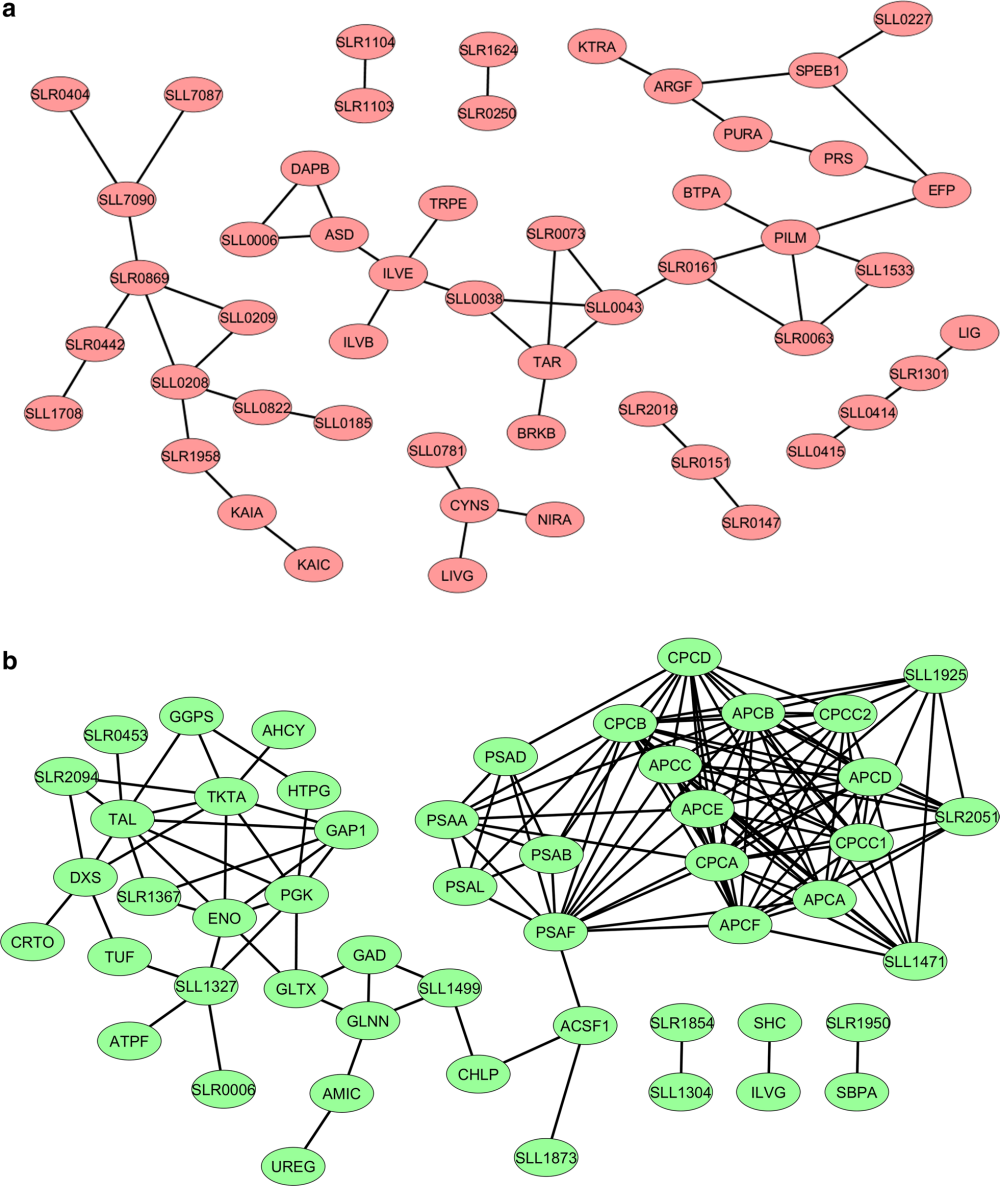
Predicted interaction of differentially expressed proteins identified in the lactate-producing mutant, SAW041. Protein interaction was analyzed and reconstructed using STRING and Cytoscape, respectively. **a** Up-regulated proteins (*α* < 0.01). **b** Down-regulated proteins (*α* < 0.01).

The distribution of the normalized protein abundance of the three biological replicates of WT, SAA012, and SAW041 is depicted in Fig. 2. As shown, the protein isotopic ratio of the WT is normally distributed around 1, further emphasizing the reproducibility of the measurements with the reference- and the WT cultures. This allows calculation of significant changes in relative protein expression level of the two product-forming strains, by using the *z* test and the protein ratio distribution of the WT as a reference, followed by Bonferroni correction. This resulted in significance thresholds for the two product-forming strains as described in: “Methods”; “Statistical analysis of the protein quantifications”. Using these boundary conditions a total number of 168 and 153 proteins, respectively, showed a significantly altered abundance in mutant strains SAA012 and SAW041. The two lists of these proteins, including their calculated *p* value, can be found in Additional file 1: Tables S4, S5, respectively. The numbers of differentially expressed proteins listed in these tables are visualized in Fig. 3. Furthermore, to establish the connection between the up-regulated proteins and the down-regulated proteins of the two product-forming strains, STRING v9.1 [34] plus KEGG pathway [35] enrichment analysis was used to predict the underlying protein interaction network. The protein interaction networks that have resulted from this analysis were reconstructed by Cytoscape v3.1.1.1 [36], and are shown in Figs. 4 and 5, for strain SAA012 and SAW041, respectively. The results of the KEGG pathway enrichment analyses showed that in SAA012, the up-regulated pathways were syn00710—carbon fixation in photosynthetic organisms, and syn00480—glutathione metabolism, while the down-regulated pathways found were syn03010—ribosome, and syn00196—photosynthesis-antenna proteins (*p* < 0.05). In contrast to SAA012, the enrichment analysis of SAW041 did not reveal specific up-regulated pathways, but only down-regulated pathways. Amongst others, photosynthesis (syn00195), photosynthesis-antenna proteins (syn00196), and glycolysis/gluconeogenesis (syn00010) were identified. An overview of the results of the KEGG pathway enrichment analyses of the two mutants is provided in Additional file 1: Table S6. Some of the observed proteins with significantly altered abundances are discussed in more detail below.

### Mutant SAA012: a high rate of ethanol production increases the overall rate of carbon fixation

Besides possible toxicity effects of the intracellular production of ethanol, we are also interested in the physiological effect(s) of rechanneling of a major part of the intermediary metabolites into our product of interest. As reported earlier [30, 31, 37], engineering a high-capacity carbon sink in a cyanobacterium is able to stimulate the overall rate of CO_2_ assimilation in such organisms. Accordingly, we have observed that our ethanol-producing strain, SAA012, showed a higher *q*_CO2_ than the WT strain ([7]; see also Fig. 1b). A remaining question is whether this higher rate of total carbon fixation is the result of an increase in the activity of ribulose-1,5-bisphosphate carboxylase (RuBisCO, e.g. by release of product inhibition) or that more RuBisCO enzyme is expressed in response to functional expression of the ethanol biosynthetic pathway (i.e. the carbon sink). The proteomic data suggest that the higher *q*_CO2_ is not exclusively a result of the up-regulation of the RuBisCO enzyme only (which is ~1.4-fold; see Additional file 1: Table S4), because some of the downstream enzymes involved in the Calvin cycle, including phosphoglycerate kinase (Pgk), fructose-bisphosphate aldolase class 1 (Fda), fructose-1,6-bisphosphatase class 1 (Slr0952), and glucose-6-phosphate 1-dehydrogenase (Zwf), that functions in pentose phosphate pathway (PPP), are also (slightly) up-regulated (see Fig. 4a). In addition, carbonic anhydrase (encoded by *slr0051*), and the proteins involved in the CO_2_ concentrating mechanism, including CcmM and CcmK1, that assist to increase the intracellular CO_2_ level, were also considerably increased. This leads to the conclusion that the CO_2_-flux in *Synechocystis* upon engineering of a carbon sink is regulated hierarchically, i.e. by changes of gene expression that adjust enzyme capacities (Vmax), rather than metabolically by interactions of enzymes with substrates, products, or allosteric effectors [38].

Significantly, we found that phosphomethylpyrimidine synthase (ThiC) which is involved in the biosynthesis of thiamine pyrophosphate (TTP) [39], was also up-regulated. Pyruvate decarboxylase (PDC) catalyses the first step in the metabolic pathway towards ethanol and is one of several enzymes that use TPP as a cofactor. An increase in TPP biosynthesis can be anticipated because of the high-level constitutive expression of the exogenous PDC [31].

### Intracellular ethanol production reduces expression of the protein synthesis machinery and the phycobilisomes, but activates an oxidative stress response

A large number of ribosomal proteins and the 10 kDa and 60 kDa chaperonins are down-regulated in the ethanol-producing strain SAA012, as is shown in Fig. 4a. This presumably is the result of the growth retardation that is observed in SAA012 (see Fig. 1a) as a consequence of the dramatic rechanneling of intermediary metabolites in these cells. Besides a reduction in the expression of the protein synthesis machinery, also the components of the phycobilisome light harvesting complex, including the structural genes CpcA, CpcB, CpcC1, CpcC2, and CpcG2 (Sll1471), as well as PbsA1 (heme oxygenase 1), HemE and HemF [i.e. the latter three all involved in chromophore (tetrapyrrole) biosynthesis], were decreased in relative abundance. Reduction in the abundance of the phycobilisome complexes in these cells was independently confirmed via recording of whole cell absorption spectra (Additional file 2: Figure S1). Significantly, none of the key functional components of the two photosystems were down-regulated in the ethanol-producing strain. A slight (0.7-fold) down-regulation was observed for Psb27, but this component has a role in PS-II repair. This may be a consequence of the reduction of the (size of the) phycobilisome antenna.

Consistent with this, the high-level ethanol-producing cells do not display an increase in their general stress response [23]. In contrast, moderate induction of the oxidative stress response as evidenced by increased levels of glutathione synthetase (GshB) and hydroperoxy fatty acidreductase (Slr1992) was observed.

### In the lactate producing mutant SAW041 a Clustered Regularly Interspaced Short Palindromic Repeats (CRISPR) system is induced

Unlike the ethanol-producing strain, the proteomics results obtained with the lactate-producing strain SAW041 did not show any specific group of proteins that was up-regulated. This includes the group of proteins identified by Kurian et al. [40] as proteins upregulated upon low-pH stress. We, therefore, tentatively conclude that significant low-pH stress is not among the consequences of redirection of a major part of the flux of carbon in *Synechocystis* towards l-lactic acid. Some of the up-regulated proteins in this strain, like DapB, Asd, ArgF and PurA, function in amino acid and nucleotide biosynthesis (see Fig. 5a). Furthermore, three proteins with unknown function, including serum resistance locus BrkB, Slr5088 and Slr0106, were observed to be strongly (i.e. >8-fold) up-regulated. Slr5088, encoded on pSYSM, was recently annotated as a short-chain dehydrogenase [41]. In the SAW041 strain, the NADH/NAD^+^ ratio is presumably decreased due to the high constitutive expression of the exogenous NADH-dependent lactate dehydrogenase (LDH). Thus, up-regulation of a dehydrogenase (i.e. Slr5088) which may be an NAD^+^-dependent enzyme, may be a consequence of this.

Besides that, three distinct proteins, i.e. Sll7065, Sll7087, and Sll7090, were found to be >2-fold up-regulated. The expression of these proteins, and their probable function, has recently been characterized [42]. They have been described as ‘CRISPR2- or CRISPR3-system associated proteins’. Significantly, two of them, i.e. Sll7065 (CRISPR2-associated protein csm3, Cas7) and Sll7087 (CRISPR3-associated protein Cmr4) were also quantified in SAA012, but did not show any up-regulation in that mutant. Sll7065 was even significantly down-regulated in the ethanol-producing strain. Sll7090 (CRISPR3-associated protein Cmr2, cas10) could only be quantified in SAW041. Little is known about the exact function of these two CRISPR systems in *Synechocystis*, except that the CRISPR3 system is the most abundantly expressed CRISPR system in WT cells [42]. The CRISPR/Cas systems are often referred to as adaptive immune systems in bacteria and archaea (see reviews [43, 44]), which confer resistance to horizontal gene transfer, including phage transduction, transformation, and conjugation [45]. It is relevant to note that the lactic acid-producing mutant has been transformed with a self-replicative plasmid, to allow for the required amount of LDH expression [30]. It is still unclear whether the CRISPR/Cas system contributes to decreased genetic stability of the SAW041 strain. Decreased production of lactic acid was occasionally observed after re-culturing this strain from a frozen stock (data not shown). An earlier study, however, on ethylene production in *Synechocystis* has reported a stable production of ethylene (>6 months) using an ethylene-producing mutant that carries a similar self-replicating plasmid [46].

### Intracellular lactic acid production reduces photosynthesis and the level of the light harvesting phycobilisome complex

The majority of the down-regulated proteins in SAW041 are involved in photosynthesis and light harvesting, as can be seen in Fig. 5a (see also Additional file 2: Figure S1; whole cell absorption spectra). Therefore, a decreasing abundance of proteins involved in central metabolic pathways, including Pgk, Tal, TktA, Eno, and Gap1, does not come unexpectedly. Whether this is cause or consequence of the slow growth phenotype observed in this strain or not cannot yet be decided. Surprisingly, however, all the ribosomal proteins quantified in this strain were not measurably changed in their abundance, which contrasts the proteomics results obtained with SAA012.

### Circadian clock components are induced by overproduction of ethanol and lactic acid

A recent proteomics study of *Synechocystis* sp. PCC6803 after treatment with excessive (i.e. much higher than the levels achievable by cyanobacterial overproduction) amounts of ethanol has suggested that the circadian rhythm of the cells may be affected by the ethanol treatment, as the circadian clock protein KaiB (Slr0757) was up-regulated [23]. In a follow-up study on the transcriptome of these cells, using the RNA-seq technique, it was shown that *slr0758,* encoding the circadian clock protein KaiC, was significantly up-regulated by ethanol stress [24]. Although KiaB was not detected in our proteomic analysis, the circadian clock proteins KaiA and KaiC were, and were both found to be up-regulated in both product-forming strains. It is still unresolved what the mechanism is behind this up-regulation and how it in turn will affect circadian physiology. Conversely, the fact that circadian physiology is important for product formation in cyanobacteria has recently elegantly been demonstrated [47]. Our results suggest that the expression of the circadian clock components may be affected by a change in (a) central metabolite(s) of the cells.

### RT-qPCR validation of the proteomic analyses

**Fig. 6:**
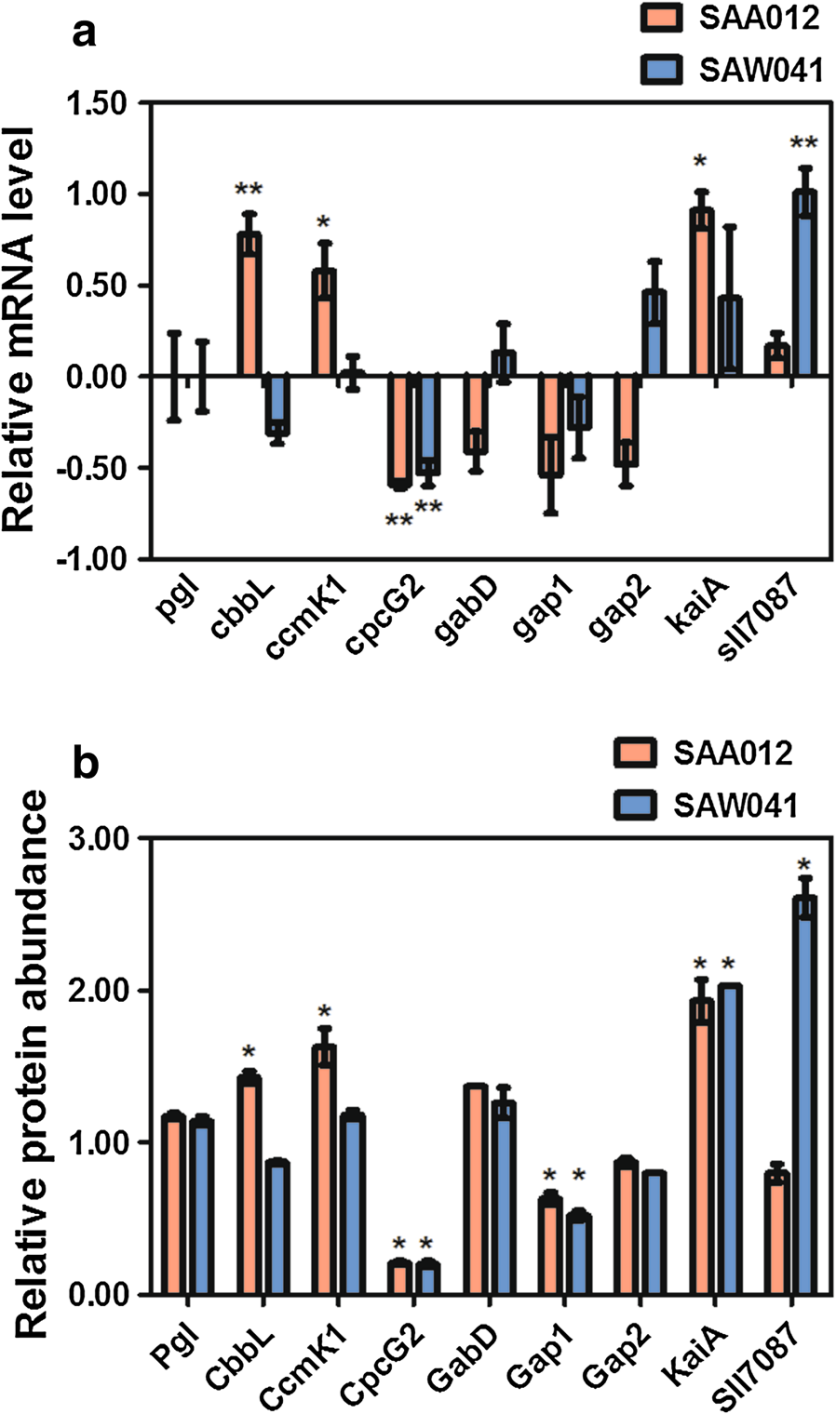
Altered mRNA- and protein expression level of selected genes of the two product-forming mutants, SAA012 and SAW041, relative to the WT strain. **a** Relative fold change of mRNA and protein level of the selected genes. Data are mean ± SEM of 3 biological- and 3 technical-replicates. Genes that were changed significantly in their level of expression are indicated with an *asterisk*. **b** Changes in relative protein abundance of the selected set of genes. Proteins with significantly changed expression level are marked with an *asterisk* (*α* < 0.01). Data are mean ± SEM of at least two biological replicates. Proteins that are shown without *error bars*, i.e. GabD in SAA012 and KaiA in SAW041, were quantified in only one biological replicate.

To verify and further characterize the significance of the quantitative proteomics results, nine genes were chosen for analysis with RT-qPCR (see Fig. 6a; “Methods”). These genes were chosen, based on their corresponding protein levels, for which a wide range of abundances was observed. This set includes genes that may play an important role in the regulation of biofuel production. Among them, three proteins did not change significantly in relative abundance, neither in SAA012, nor in SAW041 (i.e. Pgl, GabD, and Gap2). Two proteins were chosen that are similarly down-regulated (i.e. CpcG2 and Gap1) in the two mutant strains, and one protein was selected that is up-regulated in both (i.e. KaiA). Also included were CbbL and CcmK1, which were up-regulated only in SAA012, and Sll7087 (i.e. the CRISPR3-associated protein Cmr4) which was only up-regulated in SAW041.

Comparative RT-qPCR analysis between the two product-forming strains and the WT strain showed a surprisingly good correlation between the changes in mRNA expression level (Fig. 6a) and the relative protein abundance (Fig. 6b). The small SEM errors in these measurements further emphasize the accuracy of our quantitative proteomic data set and suggest that the regulatory processes involved in the responses of these selected genes operate predominantly at the level of transcription with no notable involvement of post-transcriptional regulation.

## Conclusions

Our study further demonstrates that introducing a high-capacity fermentative pathway in a cyanobacterium, i.e. an extra carbon sink that resulted in an increase of the overall rate of carbon fixation, up-regulated a set of proteins involved in the carbon concentrating mechanism, CO_2_-fixation, and the Calvin cycle, as is evident from the results obtained with the ethanol-forming strain SAA012. A similar up-regulation, however, was not observed in the lactic acid-forming strain SAW041, even though in that strain up to 50% of the fixed carbon was converted into lactic acid [30]. This is in part due to the more strongly decreased growth rate of this latter strain. Instead, we observed in this strain a strong up-regulation (i.e. ~11-fold) of Slr5088, which recently was annotated as a probable short-chain dehydrogenase [41]. This is presumably due to an imbalance of the intracellular NADH/NAD^+^ ratio, caused by the over-expressed NADH-dependent LDH. Whether the imbalance of this redox couple is also the prime reason behind the strong growth inhibition observed in this strain or not has yet to be revealed.

Furthermore, in the SAW041 strain that harbors a self-replicative plasmid, we observed a significant up-regulation of CRISPR/Cas systems (i.e. >2-fold). It is still unclear whether these systems play a role in genetic (in)stability [7, 20, 21]. It is unlikely that the CRISPR/Cas system is induced solely by an exposure to exogenous plasmid, since similar use of the self-replicative plasmid in the synthesis of 2,3-butanediol [31] and ethylene [46] did not result in a reported loss of genetic stability. Further investigations on the functional role of the CRISPR/Cas system in cyanobacteria will be necessary before its mode of action will have been resolved.

As a final point it is relevant to note many proteins that were significantly altered in their abundance (see Table 1) in the product-forming strains are either hypothetical proteins or proteins with unknown function. Thus, significantly more research on the molecular physiology of cyanobacteria is necessary for a better understanding of the mode of operation of ‘biosolar cell factories’.

**Table 1:** The top-ten most up- and down-regulated proteins quantified in at least two replica’s of the SAA012- and the SAW041 mutant strain relative to the WT *Synechocystis* sp. PCC6803, as indicated by their isotopic ratios

Protein ID	Description	^14^N/^15^N ratio		
		SAA012-1	SAA012-2	SAA012-3
P74635	Uncharacterized transporter slr0753	2.87	3.01	3.35
P73238	Slr2018 protein	2.72	3.31	2.14
P74705	Slr1178 protein		1.82	2.07
P74644	Circadian clock protein KaiA	1.78	2.07	
Q6YRW2	Slr6042 protein	1.76	1.95	
P74625	Phycobilisome rod-core linker polypeptide CpcG	0.20	0.23	0.20
P73799	Slr1259 protein	0.32		0.38
P73204	Phycobilisome 32.1 kDa linker polypeptide, phycocyanin-associated, rod 2	0.40	0.43	0.40
P73605	Slr1854 protein	0.44	0.54	0.43
P72955	Urease accessory protein UreG	0.45	0.45	0.53

Protein ID	Description	^14^N/^15^N ratio		
		SAW041-1	SAW041-2	SAW041-3
Q6ZEP2	Slr5088 protein	11.23	10.71	
Q55877	Slr0106 protein	8.77	8.63	
P73238	Slr2018 protein	2.81	3.00	2.47
Q6ZEB1	Uncharacterized protein sll7087	2.77		2.45
Q6YRT3	Slr6012 protein	2.51	2.56	2.45
P74625	Phycobilisome rod-core linker polypeptide CpcG	0.22	0.22	0.16
P73204	Phycobilisome 32.1 kDa linker polypeptide, phycocyanin-associated, rod 2	0.23	0.22	0.16
Q54715	C-phycocyanin alpha chain	0.36	0.36	0.27
P73203	Phycobilisome 32.1 kDa linker polypeptide, phycocyanin-associated, rod 1	0.44	0.39	0.28
Q54714	C-phycocyanin beta chain	0.45	0.43	0.28

## Methods

### Bacterial strains and growth condition

Wild-type *Synechocystis* sp. PCC6803, a glucose-tolerant derivative provided by D. Bhaya, University of Stanford, USA, was used as the reference strain. An engineered ethanol-producing-, and a lactic acid-producing *Synechocystis* sp. PCC6803 derivative was constructed as described previously [30, 31]. All strains were grown in triplicate in modified liquid BG-11 medium [48] supplemented with 50 mM NaHCO_3_ pH 8.0 (Sigma) at 30°C in a shaking incubator at 120 rpm (Innova 43; New Brunswick Scientific) under a constant light intensity of approximately 30 μE/m^2^/s provided by 15 W cool fluorescent white lights (F15T8-PL/AQ; General Electric) with addition of antibiotic where appropriate. For plates, BG-11 (Cyanobacteria BG-11 freshwater solution; Sigma) was supplemented with 1.5% (w/v) agar, 5 mM glucose, 0.3% (w/v) Na_2_S_2_O_2_, and antibiotic, where appropriate. To obtain a ^15^N-reference culture, modified BG-11 medium supplemented with Na^15^NO_3_ (98% atom; Sigma) instead of Na^14^NO_3_ was used. Cell density was measured with a spectrophotometer (Lightwave II, WPA). Additional information regarding the strains used in this study is provided in Additional file 1: Table S1.

### Organic acid analysis and total carbon fixation calculation

Ethanol concentrations were determined by HPLC set-up (LKB), equipped with a REZEX organic acid analysis column (Phenomenex) and a 7RI 1530 refractive index detector (Jasco). Samples were analysed at 45°C using 7.2 mM H_2_SO_4_ as the eluent. AZUR chromatography software was used for data quantification. The concentration of lactic acid was determined using the rapid assay (Megazyme), following the manufacturers’ instructions. Total carbon fixation rates, *q*_CO2_, were calculated as described previously [7] (and references therein) and shown in Fig. 1b.

### Proteomic analysis

#### Sample preparation

Cells were harvested at mid-exponential growth phase directly into an ice-cold tube with a 1/10 dilution of a Complete protease inhibitors cocktail mixture (Roche), and then centrifuged at 4,000 rpm for 10 min at 4°C. Cell pellets were immediately frozen with liquid nitrogen and stored at −80°C until use. Sampled cells of the three replicate cultures were mixed with cells from the wild-type ^15^N-reference culture at a 1:1 ratio, based on OD_730_. The mixed cell pellets were then resuspended in an extraction buffer that consisted of 6 M urea, 0.5 mM EDTA, 2% (w/v) SDS, and complete protease inhibitors cocktail mixture in 100 mM NH_4_HCO_3_ and transferred into a new tube containing 100-μm glass beads (Sigma). Cells were broken using a Precellys^®^ 24 bead beater (Bertin Technologies). Cell debris was removed by centrifugation at 15,000 rpm for 30 min at 4°C. The protein concentration of all the samples was measured by BCA assay. For further analyses 500 µg of the protein extract was reduced and alkylated using 10 mM Dithiothreitol (Sigma) and 55 mM Iodoacetamide (Sigma), respectively.

Pre-fractionation was performed using SDS-PAGE containing 10% (w/v) polyacrylamide (Bio-Rad). A total of nine protein fractions was extracted from each gel and subsequently subjected to trypsin digestion [added at a 1:10 (w/w) ratio], using an in-gel digestion method modified from [49]. The resulting peptide mixtures were lyophilized, re-suspended in 0.1% (v/v) trifluoroacetic acid (TFA) and 50% (v/v) CH_3_CN, and then loaded onto an SCX PolySULPHOETHYL Aspartamide™ column (2.1 mm ID, 10 cm length) on an Ultimate 2000 HPLC system (Thermo Scientific, Etten-Leur, The Netherlands) for cleaning purpose. Elution at a flow rate of 0.4 ml/min was performed using a 5-min linear gradient from buffer A; 10 mM KH_2_PO_4_ and 25% (v/v) CH_3_CN, pH 2.9 to B; 10 mM KH_2_PO_4_, 500 mM KCl and 25% (v/v) CH_3_CN, pH 2.9. The total peptide fraction was collected based on UV monitoring of the eluent at 214 nm. The collected peptide fraction was lyophilized and stored at −80°C. Prior to mass spectrometry analysis, samples were desalted using a C_18_ reversed phase tip (Varian).

#### LC–FT–MS/MS data acquisition, data processing and relative protein quantification

The LC–FT–MS/MS data of the 3 biological replicates per strain containing each of 9 fractions of the ^14^N, ^15^N isotopic tryptic peptide mixture were acquired with an ApexUltra Fourier transform ion cyclotron resonance mass spectrometer (Bruker Daltonic, Bremen, Germany) equipped with a 7 T magnet and a nano-electrospray Apollo II DualSource™ coupled to an Ultimate 3000 (Thermo Scientific, Etten-Leur, The Netherlands) HPLC system. The 81 samples each containing 300 ng of the ^14^N, ^15^N tryptic peptide mixture were injected as a 20 μl 0.1% (w/v) TFA aqueous solution and loaded onto the PepMap100 C_18_ (5 μm particle size, 100 Å pore size, 300 μm inner diameter, and 5 mm length) pre-column. Following injection, the peptides were eluted via an Acclaim PepMap 100 C_18_ (3 µm particle size, 100 Å pore size, 75 μm inner diameter, and 250 mm length) analytical column (Thermo Scientific, Etten-Leur, The Netherlands) using a linear gradient from 0.1% formic acid/3% CH_3_CN/97% H_2_O (v/v) to 0.1% formic acid/35% CH_3_CN/65% H_2_O (v/v) over a period of 120 min, followed by 5 min to 0.1% formic acid/90% CH_3_CN/10% H_2_O (v/v) at a flow rate of 300 nl/min. Data-dependent Q-selected peptide ions were fragmented in the hexapole collision cell at an Argon pressure of 6 × 10^−6^ mbar (measured at the ion gauge) and both precursor and fragment ions were detected in the FTICR cell at a resolution of up to 60.000 (m/Δm) with a maximum MS/MS rate of about 2 Hz. Instrument mass calibration was better than 1.5 ppm over the m/z range from 250 to 1,500. This yielded more than 9,000 MS/MS spectra over the 125 min LC–MS/MS chromatogram. Raw FT–MS/MS data of the 9 protein gel fractions were processed as multi-file (MudPIT) with the MASCOT DISTILLER program, version 2.4.3.1 (64 bits), MDRO 2.4.3.0 (MATRIX science, London, UK), including the Search toolbox and the Quantification toolbox. Peak-picking for both MS and MS/MS spectra was optimized for a mass resolution of up to 60.000 (m/Δm). Peaks were fitted to a simulated isotope distribution with a correlation threshold of 0.7 and with a minimum signal to noise ratio of 2. The processed data were searched with the MASCOT server program 2.3.02 (MATRIX science) against a complete *Synechocystis* sp. PCC6803 proteome database obtained from the UniProt consortium (May, 2014; 3,506 entries in total) plus the appropriate additional protein sequences for the two mutant strains. The database was further complemented with its corresponding decoy data base for statistical analyses of peptide false discovery rate (FDR). Trypsin was used as the hydrolytic enzyme and 1 missed cleavage was allowed in peptide identification. Carbamidomethylation of cysteine was used as a fixed modification and oxidation of methionine as a variable modification. The peptide mass tolerance was set to 10 ppm and the peptide fragment mass tolerance was set to 0.03 Dalton. The quantification method was set to the metabolic ^15^N labeling method to enable MASCOT to identify both ^14^N and ^15^N peptides. The MASCOT MudPIT peptide identification score was set at a cut-off of 20. At this cut-off, and based on the number of assigned decoy peptide sequences, a peptide false discovery rate of ~2% for all analyses was obtained. Using the quantification toolbox, the isotopic ratios for all identified proteins were determined as weighted average of the isotopic ratios of the corresponding light over heavy peptides. Selected critical settings were: require bold red: on, significance threshold: 0.05: protocol type: precursor; correction: element ^15^N; value 99.4; report ratio L/H; integration method: Simpsons; integration source: survey; allow elution time shift: on; elution time delta: 20 s; Std err. Threshold: 0.15, correlation threshold (isotopic distribution fit): 0.98; XIC threshold: 0.1; all charge states: on; Max XIC width: 200 s; threshold type: at least homology; peptide threshold value: 0.05; unique peptide sequence: on. All obtained quantification results were manually screened with the spectral data.

The MASCOT DISTILLER protein identification reports were exported as Microsoft Excel xlsx files and then imported into a custom-made VBA software program running in Microsoft Excel. The program facilitates organization and data mining of large sets of proteomics data and corrects for possible ^14^N/^15^N culture mixing errors by normalization of the isotope ratios on the median values as described elsewhere [33]. The combined protein quantification results produced accordingly are listed in Additional file 1: Table S2.

#### Statistical analysis of the protein quantifications

Significant changes in protein expression level measured in the two mutants, as compared to the wild-type strain, were calculated. A normal distribution was fitted using all the proteins measured in at least one of the three replicate WT experiments. By using this normal distribution, the significance of the change in protein expression level was calculated using the *z* test and then a Bonferroni correction was applied. Quantified proteins from the ethanol-producing strain, SAA012, with a *p* value <1.21E−05 was considered as a significant change (*α* < 0.01), while 1.14E−05 was used as the threshold for significant change (*α* < 0.01) in quantified proteins from the lactic acid-producing strain, SAW041. The small difference in threshold value between the strains is caused by the fact that not the same number of proteins is identified in both strains.

### RNA isolation and real-time quantitative PCR

Cells were harvested at the mid-exponential growth phase and then centrifuged at 4,000 rpm for 10 min at 4°C. Cell pellets were immediately frozen with liquid nitrogen and stored at −80°C until use. Cells were opened using a Precellys^®^24 bead beater and cell debris was removed by centrifugation. RNA was isolated using the RNeasy mini kit (Qiagen). The concentration of RNA was measured using a Nanodrop 1000 spectrophotometer (Thermo Scientific), while RNA quality was determined using 1% agarose gels. The RevertAid First Strand cDNA Synthesis Kit (Thermo Sciecntific) was used to synthesize cDNA. Quantitative reverse transcription-PCR (RT-qPCR) was performed with cDNA in an Applied Biosystems 7300 Real Time PCR system using Power SYBRs Green PCR Master Mix (Life Technologies). Primers were designed using Primer3 software (Life Technologies) and are listed in Additional file 1: Table S3. The relative mRNA levels of eight selected genes, i.e. *cbbL*, *ccmK1*, *cpcG2*, *gabD*, *gap1*, *gap2*, *kaiA*, and *sll7087*, were calculated using the ∆∆CT method [50], and normalized using *pgl* as the internal control. The statistical significance level was calculated using the *t*-test and corrected by the Bonferroni method.

## Additional files

Additional file 1:
**Table S1.** Strains used in this study. **Table S2.** A total of 1039 normalized protein ^14^N/^15^N isotopic ratios including their mascot scores from 3 biological replicates quantified in *Synechocystis* sp. PCC6803 wild-type (WT) strain, ethanol-forming strain (SAA012) and lactic acid-forming strain (SAW041). **Table S3.** List of primers used in RT-qPCR. **Table S4.** List of proteins quantified in SAA012 that were signifcantly changed and its *p*-value from *z*-test. **Table S5.** List of proteins quantified in SAW041 that were signifcantly changed and its *p*-value from *z*-test. **Table S6.** Results of KEGG pathway enrichment analyses of the significantly altered proteins quantified in the ethanol-producing strain (a) and the lactic acid-producing strain (b). Additional file 2:
**Figure S1.** Whole-cell absorption spectra of *Synechocystis* sp. PCC6803 wild-type strain, ethanol-producing strain (SAA012), and lactate-producing strain (SAW041) at mid-exponential growth phase.

## Abbreviations

Cas: CRISPR-associated genes
CRISPR: clustered regularly interspaced short palindromic repeats
DW: dry weight
FDR: false discovery rate
FT and FTICR: Fourier transform ion cyclotron resonance
LC: liquid chromatography
L/H: light/heavy
LDH: lactate dehydrogenase
MS: mass spectrometry
MudPIT: multidimensional protein identification technology
PDC: pyruvate decarboxylase
RuBisCO: ribulose-1,5-bisphosphate carboxylase/oxygenase
SDS: sodium dodecyl sulfate
SEM: standard error of the mean
TFA: trifluoroacetic acid
TTP: thiamine pyrophosphate
WT: wild-type
XIC: extracted-ion chromatogram
