# Supplementary material for: Quantitative proteomics analysis of an ethanol- and a lactate-producing mutant strain of Synechocystis sp. PCC6803
Source: Biotechnol Biofuels. 2015 Aug 5;8:111. doi: 10.1186/s13068-015-0294-z (PMC4526308; doi:10.1186/s13068-015-0294-z)

Additional file 2: Figure S1. Whole-cell absorption spectra of *Synechocystis sp.* PCC6803 wild-type strain, ethanol-producing strain (SAA012), and latate-producing strain (SAW041) at mid-exponential growth phase.

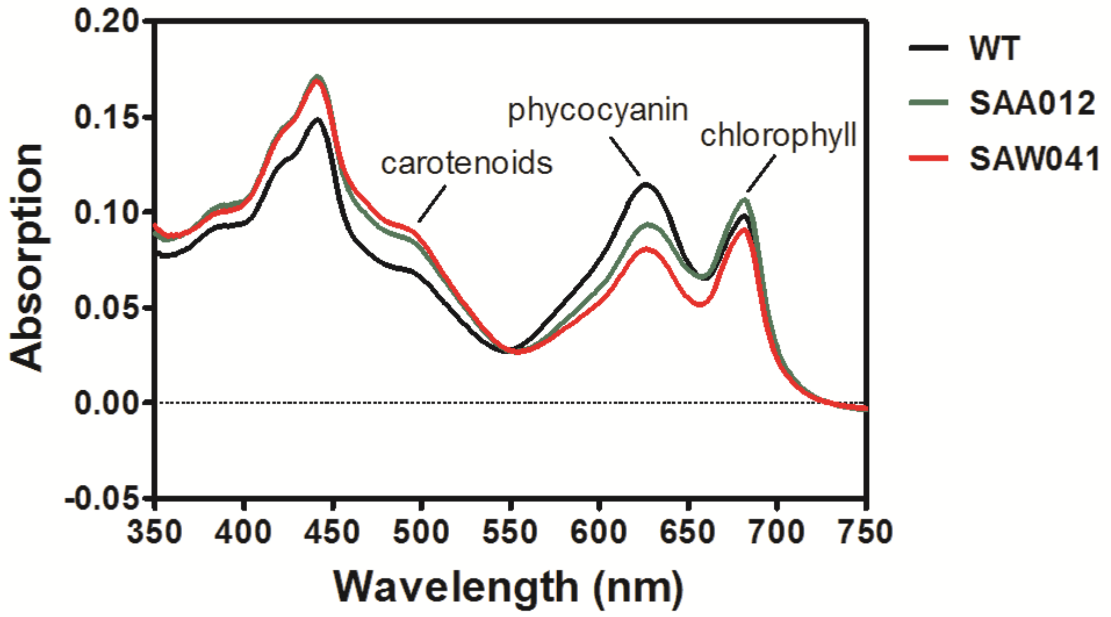

Supplement: Additional file 2: — Figure S1. Whole-cell absorption spectra of Synechocystis sp. PCC6803 wild-type strain, ethanol-producing strain (SAA012), and lactate-producing strain (SAW041) at mid-exponential growth phase. [file 13068_2015_294_MOESM2_ESM.pdf]
